# Return Migration among Elderly, Chronically Ill Bosnian Refugees: Does Health Matter?

**DOI:** 10.3390/ijerph121012643

**Published:** 2015-10-12

**Authors:** Line Neerup Handlos, Karen Fog Olwig, Ib Christian Bygbjerg, Maria Kristiansen, Marie Louise Norredam

**Affiliations:** 1 Danish Research Centre for Migration, Ethnicity and Health, Section for Health Services Research, Department of Public Health, University of Copenhagen, Øster Farimagsgade 5, Copenhagen K 1014, Denmark; 2 Department of Anthropology, University of Copenhagen, Øster Farimagsgade 5, Copenhagen K 1353, Denmark; E-Mail: Karen.fog.olwig@anthro.ku.dk; 3 Section of Global Health, Department of Public Health, University of Copenhagen, Øster Farimagsgade 5, Copenhagen K 1014, Denmark; E-Mail: Iby@sund.ku.dk; 4 Center for Healthy Aging, Section for Health Services Research, Department of Public Health, University of Copenhagen, Øster Farimagsgade 5, Copenhagen K 1014, Denmark; E-Mail: Makk@sund.ku.dk; 5 Danish Research Centre for Migration, Ethnicity and Health, Section for Health Services Research, Department of Public Health, University of Copenhagen, Øster Farimagsgade 5, Copenhagen K 1014, Denmark; E-Mail: Mano@sund.ku.dk; 6 Section of Immigrant Medicine, Department of Infectious Diseases, University Hospital Hvidovre, Hvidovre 2650, Denmark

**Keywords:** return migration, elderly, chronic illness, well-being, Bosnia and Herzegovina

## Abstract

Elderly migrants constitute a considerable share of global return migration; nevertheless, literature on the health aspects of the return migration among these migrants is still scarce. This study explores the significance of return migration among elderly, chronically ill Bosnian refugees from Denmark and the role of health issues in their decision to return. It is based on semi-structured interviews with 33 elderly, chronically ill Bosnian refugees who have moved back to Bosnia and Herzegovina, and 10 elderly, chronically ill Bosnian refugees who have remained in Denmark. The interviews show that physical health, in the sense of the absence of illness and easy access to necessary health-care services and medicines, was not highly prioritized when the decision was made whether or not to return. However, if health is regarded more broadly as involving more than mere physical health and the absence of illness, health did matter. Viewed as physical, social and mental well-being in line with WHO’s definition of health, health was indeed one of the most important factors when the decision to return was made.

## 1. Introduction

People have always migrated in search of new opportunities or in order to escape conflict or poverty. With time, migration has become easier and people have become more mobile. Concomitantly, transnationalism, in terms of people having professional and private ties to institutions and individuals in various countries simultaneously, has become more widespread. With transnationalism a definitive end has been put to migration as a uni-directional and permanent movement of people, and circular movements, including return migration to the country of origin, have become common [1]. 

Motives for return migration to the country of origin have been found to vary considerably. Among Turkish labour migrants in Germany, value-oriented reasons such as the desire to live in a society with Turkish culture, a fear of losing Turkish culture abroad and concerns over the allegedly unhealthy and stressful German life-style and the bad climate were found to be the main drivers for return migration [2]. Another study found that, among all nationalities of migrants living in Denmark, out-migration, including return migration, was partly determined by the level of one’s economic success in Denmark: where migrants experienced limited success in the Danish labour market, the propensity to return to their country of origin was higher. However, family ties, in particular the number of children living in Denmark and the nationality of the spouse, were also important factors influencing whether or not people decided to return [3]. Nonetheless, a study of intentions to return among Moroccan migrants living throughout Europe showed that labour market participation in the receiving country did not predict intentions to return, but that the effectiveness of socio-cultural integration did [4]. The literature thus indicates that social and cultural factors generally play an important role when decisions concerning return migration are made. 

The ageing of the global population has had an impact on the characteristics of migration worldwide, meaning that many migrants, including return migrants, are now elderly [5]. Nevertheless, literature on the health aspects of return migration among elderly migrants is still scarce [6,7]. Ageing is often accompanied by chronic illness, and the share of migrants who are chronically ill is therefore also increasing. Chronically ill individuals need continuous treatment and are dependent on frequent contact with the health-care system. Furthermore, elderly people are generally physically vulnerable and they are prone to develop more diseases as they age [8,9]. One would therefore expect that elderly, chronically ill people would prioritize being near good health-care facilities. However, this is not always the case. 

The present article explores drivers among one of the ethnic groups that has recently undertaken return migration out of Denmark, namely elderly, chronically ill Bosnians [10]. More specifically, the article explores the following questions: (1) why do Bosnian refugees return to Bosnia when they are old and ill? And (2) what role do health issues play in connection with the decision to return? 

### Context

In order to understand the drivers behind return migration to Bosnia and Herzegovina (hereafter referred to as Bosnia), it is necessary to examine the context in which migration initially occurred, as well as the context in which the return takes place. Bosnia gained independence from Yugoslavia in 1992. This started a war that lasted three years and resulted in 2.2 million people fleeing their homes, 1 million of whom left the country [11]. People of all age groups fled. The majority went to Germany, but many others went to Austria, the Netherlands and Sweden [12]. Around 17,000 fled to Denmark, where they were all eventually granted asylum [13].

When the war ended, an important part of the international community’s strategy for the reconstruction of Bosnia was to repatriate as many Bosnian refugees as possible in order to neutralize the effects of the ethnic cleansing that had taken place during the war [14]. The repatriation policy was to a large extent based on the assumption that return migration is desirable for all refugees and that re-integration into one’s home country happens naturally and quickly [15]. 

Despite massive efforts to repatriate Bosnian refugees, by 2015 only around 1 million of the 2.2 million refugees had returned to their pre-war place of residence [11]. From Denmark, 2705 individuals had returned by the end of 2014 (this figure represents the number of Bosnians who have left Denmark; from the Danish Refugee Council’s repatriation registry, we know that the majority of these individuals have repatriated to Bosnia, however, the number of return migrants is probably slightly lower than the figure presented here, as some people have out-migrated to countries other than Bosnia). Around 900 returned within the two years following the ending of the war, most of them being relatively young people below the age of forty. The annual number of return migrants decreased throughout the first decade of the 21st century, but grew again in 2010, when the Danish state increased the support given to repatriating refugees (see below). Most of these returnees were elderly people between the ages of 60 and 80 [10]. 

The current situation in Bosnia is characterized by political instability involving problems with democratization, inter-ethnic cooperation and the protection of minority rights. The standard of living is among the lowest in Europe, the unemployment rate is high, corruption is widespread, and many public-sector employees and people on welfare payments are not being paid on a regular basis [16,17,18]. With regard to health care, and specifically the availability of continuous treatment for chronic illness, in 2001 the Office of the United Nations High Commissioner for Refugees (UNHCR) stated: “It is evident that it may not be possible for patients with chronic disease to obtain the necessary treatment in the territory of Bosnia and Herzegovina. At the current levels of treatment, the lives of persons in need of medical treatment for chronic diseases or conditions … may be jeopardised if they are forced to seek treatment in Bosnia and Herzegovina” [19]. Other recent literature also concludes that treatment options in Bosnia are not adequate and that the health-care system does not function well [20].

The Bosnian refugees who came to Denmark as children or adolescents are often described as well-integrated. Many have acquired higher education, and the majority possess good jobs. By contrast, few of those who came to Denmark as adults managed to learn Danish or obtain waged employment. Now, twenty years after their escape from Bosnia, many of those above the age of fifty still have limited contact with employment or civil society in Denmark [21,22]. 

Return migration among individuals from Bosnia is voluntary and is supported financially by the Danish state with a one-off payment, medical support for the first year and lifelong reintegration payments to everyone above 55 years of age. The size of the one-off payment increased considerably in January 2010, as did the size of the monthly payments, the former now constituting approximately 17,000 Euros and the monthly payments 470 Euros/month for five years or 375 Euros/month for life. In comparison, the average monthly salary in Bosnia is 363 Euros (gross national income *per capita* as reported by the World Bank 2014 [23]). The returnees give up their Danish residence permit when returning to Bosnia; however, during the first year of their return they are allowed to change their minds and return to Denmark to reclaim their residence permit [24]. Only very few have used the right to do so (personal communication [25]). 

## 2. Methodology

In order to explore the drivers for return migration among elderly, chronically ill Bosnians, both Bosnian return migrants and Bosnians who had decided to stay in Denmark were interviewed. The criteria for inclusion in the study were age above 55 years and a diagnosis of a chronic illness. The interviewees who resided in Bosnia were recruited with the assistance of a repatriation consultant at the Danish Refugee Council and informants who had already been interviewed, using the snowballing method. The consultant had information on individuals’ illness statuses. In Denmark various Bosnian community centres were approached, and recruitment was done through these as well as through snowballing. 

The first author performed semi-structured interviews with 33 chronically ill and elderly Bosnians who had returned from Denmark to Bosnia, and 10 chronically ill and elderly Bosnians who continued to live in Denmark. Furthermore, three health-care professionals, of whom one resides in Denmark and two in Bosnia, were interviewed for background information. The interviews in Bosnia were conducted in cities and villages, all of them in the homes of the interviewees. The majority of the interviews in Bosnia were conducted with married couples. Interviewing the husband and wife together had several advantages. These couples were elderly and had spent most of their lives together, including their history of flight and refuge in Denmark, and they were therefore very close and shared many experiences and opinions. They did most of their activities in each other’s company, and the interviewer was greeted as a guest in their home by both. Hence, insisting on interviewing individuals alone would not have been socially appropriate. Furthermore, the decision to return to Bosnia appeared to be very much something they had discussed thoroughly with each other and it therefore added nuance to the description that they were both present during the interview. The interviews in Denmark were with individuals, not couples, as they were either carried out with single persons or conducted in a community centre to which only men came.

All interviews were conducted during the winter and spring of 2013/2014 with the assistance of Danish–Bosnian interpreters. The interviews lasted between 45 minutes and 3 hours. Even though some interviews lasted longer than others, the point at which no new information on the topic of investigation was revealed was generally reached before the interview was completed. All interviews were transcribed verbatim and translated into Danish. The analyses were inspired by systematic text condensation as described by Malterud [26] using Nvivo, a tool for analyses of qualitative data. This method permits an explorative approach to the data and is not theory-based as such. Themes which were relevant for explaining health issues in motives for return migration were identified, and quotes which substantiated and illustrated key points were selected and translated into English. Relevant theory on transnationalism, inter-generational caretaking, belonging and the physical aspect of the migration process were drawn upon in the ethnographic analysis. 

### 2.1. Ethics

An interpreter informed all interviewees by phone or by letter and phone about the purpose of the study before they were asked to agree to meet the interviewer. In accordance with the American Anthropological Association’s ethical guidelines, a more thorough introduction to the study, including the interviewee’s right to withdraw from it at any time, was given to each individual before the actual interview took place [27]. All potential participants, except for two, gave final consent to participate. Consent was given verbally. Much consideration was given to the differences related to cultural background, gender and age between interviewer and interviewees. Guided by the interpreter of Bosnian origin, the interviewer paid attention to and showed respect for social codes (for example, by addressing the informants in a respectful way, by letting the informants talk about topics they wanted help with or topics they wanted the interviewer to know about, and by accepting food and beverages offered). After the interview, a small present was given to the interviewees as a symbol of gratitude. All informants were ensured anonymity. 

### 2.2. Characteristics of Informants 

The characteristics of the informants are shown in Table 1 and Table 2. All informants had fled from Bosnia to Denmark during the 1990s; none had immigrated to Denmark as labour migrants. Of those residing in Bosnia at the time of the interview, one couple and one man had returned respectively ten and eleven years prior to the interview, whereas the rest had returned less than four years beforehand. Except for one individual who was not ill, all informants were suffering from one or more of the following chronic diseases: type 1 or type 2 diabetes, cardiovascular disease (hypertension or a history of angina pectoris, heart failure or thrombosis), asthma, depression, post-traumatic stress disorder (PTSD), chronic pain, arthritis, epilepsy, chronic diarrhoea or psoriasis. At the time of the interview all informants were retired or unemployed, and very few had been employed while living in Denmark. 

**Table 1:** Characteristics of informants residing in Denmark.

Participant Number	Age *	Sex	Living with Children	Living in the Same Country as Children	Illness	Educational Level **	Employment in Bosnia before the Flight	Employment in Denmark
1	1	Male	No	Yes	Diabetes and CVD	2	Constructor	None
2	1	Male	No	Yes	Diabetes and CVD	2	Accounts manager	Accountant
3	1	Male	No	Yes	None	3	Engineer	Teacher
4	2	Male	No	Yes	CVD	2	Chef	None
5	1	Male	No	Yes	Diabetes, epilepsy	2	Carpenter	None
6	2	Male	No children		Fibromyalgia	2	Teacher	Teacher
7	1	Male	No	Yes	Asthma	2	Installation worker	None
8	1	Female	No	Yes	Diabetes	2	Referent	None
9	1	Female	No	Yes	Chronic pain	1	Nursery teacher	None
10	3	Male	No	Yes	CVD	Unknown	Unknown	Unknown

* Age in years: 1 = 55–65, 2 = 66–75, 3 = 76–85; ** Educational level: 1 = primary and lower secondary school, 2 = youth education, 3 = higher education; CVD: cardiovascular disease.

**Table 2:** Characteristics of informants residing in Bosnia.

Participant Number	Age *	Sex	Years since Return	Living with Children	Living in the Same Country as Children	Illness	Educational Level **	Employment in Bosnia before the Flight	Employment in Denmark	
11	3	Female	1	No	Yes	CVD	2	Cashier	None	
12	3	Male	1	No	Yes	CVD	2	Electrician	None	
13	1	Female	2	Yes	Yes	Diabetes, asthma	1	Housewife	None	
14	2	Male	2	Yes	Yes	Diabetes	2	Workman	None	
15	3	Male	2	Yes	Yes	Psoriasis	2	Electrician	None	
16	1	Female	3	Yes	Yes	Chronic pain	1	Housewife	None	
17	1	Male	3	Yes	Yes	Chronic pain	2	Barrel maker	None	
18	2	Female	4	No	Yes	Diabetes	Unknown	Housewife	Unknown	
19	2	Male	4	No	Yes	CVD	Unknown	Odd-job man	Unknown	
20	2	Female	10	No	Yes	CVD	2	Finance manager	None	
21	1	Male	10	No	Yes	Diabetes	2	Purchasing agent	None	
22	2	Female	1	No	Yes	CVD	1	Housewife	None	
23	2	Male	1	No	Yes	Chronic pain	1	Mechanic	None	
24	1	Female	2	No	Yes	Chronic pain	2	Seamstress	Nursery teacher	
25	2	Male	2	No	Yes	PTSD	2	Office worker	None	
26	2	Female	4	No	Yes	Chronic pain	1	Housewife	None	
27	2	Male	4	No	Yes	Chronic pain	1	Labourer	None	
28	2	Female	1	Yes	Yes	Diabetes	1	Chicken farmer	None	
29	2	Male	1	Yes	Yes	Chronic pain	1	Bricklayer	None	
30	3	Female	3	No children		Chronic diarrhoea	1	Housewife	None	
31	3	Male	3	No children		CVD, Parkinson’s disease	2	Fireman	None	
32	2	Female	3	Yes		Yes	Diabetes	1	Cashier	None
33	2	Male	3	Yes		Yes	PTSD	1	Mechanic	None
34	1	Female	4	No		No	Diabetes	1	Referent	None
35	1	Male	4	No		No	Chronic pain	1	Shop manager	None
36	3	Female	3	Yes		Yes	Diabetes	1	Housewife	None
37	1	Female	3	No		Yes but deceased	None	1	Housewife	None
38	2	Male	3	No		Yes but deceased	Diabetes	1	Manager	None
39	3	Female	3	No		Yes	Diabetes and arthritis	1	Housewife	None
40	3	Male	11	No		Yes	CVD	1	Butcher	None
41	2	Male	1	No		Unknown	Diabetes	Unknown	Carpenter	None
42	2	Female	3	No		No	Diabetes	Unknown	Housewife	None
43	3	Female	3	No		Yes	CVD and arthritis	2	Laboratory technician	None

* Age in years: 1 = 55–65, 2 = 66–75, 3 = 76–85; ** Educational level: 1 = primary and lower secondary school, 2 = youth education, 3 = higher education; CVD: cardiovascular disease; PTSD: post-traumatic-stress disorder. The following participants were interviewed in dyad interviews: 11 and 12, 13 and 14, 16 and 17, 18 and 19, 20 and 21, 22 and 23, 24 and 25, 26 and 27, 28 and 29, 30 and 31, 32 and 33, 34 and 35, 37 and 38, 39 and 40.

## 3. Findings

In the following, findings from the interviews are presented focusing on the reasons why Bosnian refugees return to Bosnia when they age and become ill and on the role that health issues play in the decision to return migrate.

### 3.1. Considerations Related to Access to Health-Care Services

In accordance with the previously mentioned assessment of the Bosnian health-care system’s capacity to treat chronic illness, the informants described access to health care and cost of medicine as being unfavourable in Bosnia compared to Denmark. One married couple (Participants 37 and 38) who had returned to Bosnia explained that they spent 60% of their income on the husband’s diabetes medicine, whereas in Denmark most of their medical expenses had been covered by the state. Besides considerable expenses, several informants also mentioned how access to health-care services in Bosnia was obstructed by long waiting lists and corruption in the system. Many informants explained that they had to bribe health-care professionals in order to obtain the appointments they needed. As one woman said:

Over there (in Denmark) we had everything. Here you pay for everything.

(Participant 37)

However, the interviewees did not describe access to the Danish health-care system as unproblematic: language barriers and a lack of available interpreters were cited as factors contributing to communication complications with health-care professionals. This was mentioned as being a great problem by some of the informants. A few informants preferred the type of medical treatments available in Denmark, but most found the quality of treatments in Denmark and in Bosnia to be relatively similar.

Despite medical treatment being described as more easily available in Denmark than in Bosnia, only a minority of the interviewees residing in Denmark mentioned barriers in accessing health care in Bosnia as one of the reasons why they had chosen to stay in Denmark; the vast majority had not prioritised easy and inexpensive access to health care when deciding where to live. The case of one informant, whose illness worsened upon his return to Bosnia due to the unavailability of medication, illustrates how access to treatment was often not prioritized when the decision to return was made:

Interviewer: *If you had known before you returned that your psoriasis would worsen or spread, would you then have stayed in Denmark?*

Informant: *We did not want to stay (in Denmark).*

(Participant 15)

The decision to return was very well-informed for most informants with regard to the advantages and disadvantages for their health status and access to health care; they had considered things thoroughly. When a couple who had returned to Bosnia and who were suffering from chronic pain and PTSD respectively were asked about their concerns on returning to a health-care system with less easy access, the husband replied:

We thought about it (access to health care in Bosnia), we wrote everything down on a piece of paper. In the end we decided to return. We knew everything.

(Participant 25)

So, did people deliberately not prioritise their health when they decided to return? If optimization of health is regarded as pursuing good physical health and good access to health-care services, it can be concluded that in general the interviewees were driven by other factors when they decided where to reside. On the other hand, if health is regarded as a broader notion that involves more than physical health and access to health care—as implied by the World Health Organization (WHO)’s definition: “complete physical, mental and social well-being and not merely the absence of disease or infirmity” [28]—one could argue that the returning Bosnians did indeed prioritize health when they made their decision, as they prioritized factors that would give them better physical, mental and social well-being. 

A quote from a returnee couple who both suffered from chronic illness illustrates how deliberate the decision to choose well-being in a country with lower levels of welfare and prosperity had been for them:

I’m fine with just eating once a day, as long as I am here.

(Participant 37)

Instead of eating five times somewhere else, we prefer to eat once here.

(Participant 38)

The following sections in this paper will explore the significance of general well-being further by examining the relationship between return migration and the physical, mental and social aspects of well-being. Even though the aspects are intrinsically interconnected and therefore not necessarily meaningful on their own, we will attempt to disentangle them in order to acquire a deeper understanding of this issue. As the questions on the motives for return migration were open ones, informants were able to define themselves what was important in their decision. The three aspects presented in this article were mentioned in almost all interviews. 

### 3.2. Physical Well-Being 

The physical aspect of well-being related to return figured as an important consideration when people decided where to reside. One informant, who suffered from PTSD and who had experienced many violent and upsetting things in Bosnia but nevertheless had returned after twenty years in Denmark, explained his decision as follows:

Interviewer: *Why are you so happy to be back (in Bosnia)*?

Informant exhales deeply and smiles.

Interviewer laughs.

Informant: *Something like that, but it is hard to compare. But let’s imagine I am sitting on a chair that is shaking and I then move to a chair that is solid; it’s that inner feeling.*

(Participant 25)

A feeling of general physical well-being accompanied the return for many of the informants. Factors such as the climate and the physical environment were often described as decisive in drawing informants back to Bosnia. One man who had returned with his wife three years prior to the interview and who had a history of several heart operations and Parkinson’s disease described what had made him return:

What I have here (in Bosnia) is a nice atmosphere. The air is good, the water is good, the water from the spring is clean. Here I have flowers. Every morning I go out and exercise. I breathe. When I was in Denmark I had the apartment, the balcony. I only looked to the left and to the right. I didn’t have anything else.

(Participant 31)

The couple experienced great financial difficulties after their return to Bosnia, and they had no children who could support them, so they faced even harder times ahead. However, well-being related to the physical aspects of being in Bosnia compensated for the difficulties they encountered, and they were happy with their decision. 

Nearly all informants said that, despite having less material wealth and worse access to health care, they felt better and more content after having returned to Bosnia. Often any possible deterioration of physical health that had happened after the return was explained by the ageing process and seen as being countered by an increase in contentment and well-being. As one returnee said:

*When it comes to illnesses, I age every day. The older you get, the more ill you become. But overall, the sun is shining on me. Even if I have less, I am more content here (in Bosnia).*

(Participant 38)

### 3.3. Social Well-Being

Another important dimension of well-being mentioned in the interviews was the social aspect. Many of the interviewees had family members in different parts of the world. They lived somewhat transnational lives; visits to family members in various places were common, as were long vacations in Bosnia. Despite the frequent visits to see family, nearly all the informants who had returned to Bosnia mentioned their desire to be with their children as the main reason for their return. The children had, for political reasons, often returned before the parents. Many of them had fled the war earlier than the parents and went to Germany, where there was already a large population of Yugoslav labour migrants. By the time the parents had fled the war, Germany had closed its borders to refugees from the Balkans. This forced the parents to go elsewhere, among other places to Denmark. When the war ended all Bosnians were expelled from Germany, and the children therefore returned to Bosnia, whereas those who had been granted asylum in Denmark stayed there. As seen in Table 2, only one of the returning couples and one of the returning singles did not have children in Bosnia (apart from one couple who had no children). The fact that all of the informants who resided in Denmark had children who lived in Denmark (apart from one informant who had no children) further stressed the importance of physical proximity to one’s children (data presented in Table 1). It seemed to be a matter of course that the informants wanted to be with their children. As noted by one man who had children in Bosnia and who had lost his wife when he returned to Bosnia:

If my children still lived in Denmark I wouldn’t have returned… You follow the children... We didn’t want to stay (in Denmark)… Because of the children we couldn’t stay at all. You know how it is.

(Participant 15)

Another man who lived in Denmark and whose son also lived there put it this way:

I thrive here (in Denmark) because of my son. I love Denmark because of my son.

(Participant 4)

The importance of being with one’s children as a motive for return migration was stressed by the fact that several of the informants who had returned to Bosnia had settled where their children were living, not where they themselves had been living when they fled. 

In some cases, where parents had more than one child, the children lived in different countries. This compelled the parents to choose which child to live near, and this was usually determined by weighing who had the greatest need for their parents. Often children who did not have a spouse or children who were sick and whose family therefore needed help were given the highest priority. One of the interviewed couples had two children, one living in Sweden and the other in Bosnia. As their son who lived in Bosnia was ill, they decided to return there, though he died shortly after their return. In explaining why they had returned, they emphasized that his death had confirmed the appropriateness of their decision, as they were now able to support their surviving relatives. As the woman said:

At least we can live with the grandchildren, side by side with his children.

(Participant 37)

The couple also explained that being physically near their son’s grave was very important to them and gave them a sense of being near him. 

The informants who had returned to Bosnia described how they had missed their children deeply while living in Denmark and how being near them and their grandchildren gave them peace of mind. Some informants mentioned how they valued having a role in their children’s lives again, among other things by helping them take care of the grandchildren. Being able to help their children and being close to their grandchildren seemed to give joy and meaning to informants’ lives. It was something they had longed for. 

Both the returnees and those who stayed in Denmark explained how being near the children and grandchildren also provided them with assistance with household chores such as cleaning and cooking, as well as assistance in case of emergencies. As one woman who had returned to Bosnia and now lived in the same house as her children said:

When something hurts or I am in a critical state, then my children are here. They immediately call the ambulance.

(Participant 13)

Another woman who had returned to Bosnia explained how her grandchild who lived nearby helped her and her husband:

Our grandchild visits us, calls on us. She visits, buys bread for us, picks up cigarettes and stuff like that. She also buys medicine for us. They are good kids, really good.

(Participant 37)

Similarly, a man who lived in Denmark described how he relied on assistance from his son in many ways:

Well, it’s a good thing that I have my children, because it’s really difficult to be in a foreign country with a foreign language. If we need help, my son comes over and drives us to the doctor and fixes everything. I don’t know what I would have done if it hadn’t been for him.

(Participant 5)

The fact that the informants had a chronic illness and were facing old age with an expected loss of functionality emphasized their need for assistance with practical matters and health-care services, and thus their need to be with their children. One woman, who had serious cardiovascular problems and had returned with her husband one year prior to the interview, simply stated that:

*The older you get, the more you need your children.*

(Participant 23)

A childless couple who had returned to Bosnia illustrate how difficult it is to live without children in Bosnia as an elderly, chronically ill person:

Nobody can help us; we have no children. It’s hardest now. I have suffered a lot, but now is the hardest time. In Denmark, if you have no children, you have insurance, isn’t that so?

(Participant 30)

The need for assistance from children also reflects the fact that no real alternative method of obtaining assistance existed for many, as they could not afford elderly care and nursing homes in Bosnia. 

The prospect of spending one’s old age in a nursing home in Denmark was another reason for the interviewees to return to Bosnia. As one woman, who at the time of the interview was residing in Denmark, said:

If I have to go into a nursing home, I’d prefer to stay in a nursing home in Bosnia. Because I know the language they speak. I do understand some Danish, but I would move to Bosnia. … What would I be doing at a nursing home here?

(Participant 8)

Several other informants expressed an unwillingness to stay in Danish nursing homes. They mentioned the language barrier as the main reason why these homes were so undesirable. However, while Danish nursing homes were very unpopular, nursing homes in general were disliked. One man who had returned to Bosnia explained why:

Informant: *We don’t want to go into a nursing home. It would be embarrassing to go into a nursing home when we have family.*

Interviewer: *Embarrassing for your children or for you?*

Informant: *Embarrassing for our children. If the neighbour, for example, heard about it, they would right away say that the children were bad children.*

(Participant 23)

Altogether, being near the family and having a role in it gave the informants social and—as appears from the following section—mental well-being. 

### 3.4. Mental Well-Being 

One final aspect of well-being which was also important for why the elderly, chronically ill Bosnians interviewed in the present study returned was their mental well-being. Informants linked mental well-being closely to a feeling of belonging, and they expressed in various ways how the pursuit of belonging had made them return. One man who had returned to Bosnia said:

Nowhere but here (in Bosnia) do I feel at home. I was born here, I have lived here. If I had felt the same way in Denmark, I would have stayed in Denmark and not returned.

He continued by quoting a local song: *You can go everywhere, but you will always return.*

(Participant 35)

One woman (Participant 36) who had returned to Bosnia described how she cried when she saw young people in Denmark because, as she said, “*they were so lucky to be at home”*. Seeing them being where they felt they belonged and where they felt at home made it clear to her that she did not feel at home in Denmark, and that made her deeply unhappy. 

It seems that ageing emphasized the desire to be where one belonged. One woman who had returned, despite her traumatic experiences during the war, said:

The older you get, the more you want to go where you belong.

(Participant 28)

Many described how they always expected to spend their old age in Bosnia and how they had regarded it as a matter of course that they would return at some point. It was a matter of following one’s fate, but also a matter of returning before it was too late. Some mentioned that they had focused on returning while they were still alive so they would spare their relatives the hassle of bringing a corpse back to Bosnia in a coffin:

We decided to return so they wouldn’t have to tow me; in this way I came in a wheelchair.

(Participant 12)

Connected to this was the fact that many of the informants did not want to die in Denmark; it was important that they died and were buried in Bosnia alongside their ancestors. As a man who had returned said:

All our relatives are buried at the graveyard here, so we are next.

(Participant 27)

Besides the desire to be close to their ancestors in the grave, there was also a strong desire to be close to the graves of relatives while they were still alive; the family bonds extended beyond death. In many cases, family members who had died in Denmark had been sent to Bosnia to be buried. An additional explanation is that Danish and Bosnian burial traditions differ in terms of religious and cultural practices [29]. 

The fact that these informants—unlike labour migrants—had been forced to migrate also had an impact on where they felt they belonged and why it was so important to return. As one man who had returned to Bosnia, but subsequently had had to return to Denmark again, put it:

I returned because of a desire to go back to my own country; I didn’t come to Denmark because I wanted to.

(Participant 6)

Another informant (Participant 9) residing in Denmark said that the fact that she did not choose to leave Bosnia made her feel that something in her life was unfinished. This informant had other reasons not to return (she had been abused during the war and was afraid of meeting the offenders in Bosnia), but she very strongly felt the need to do so. 

The interviews thus showed that the returned informants had obtained higher levels of physical, social and mental well-being by returning to Bosnia. This was supported by the fact that none of the informants who had returned regretted doing so. 

## 4. Discussion

Migration can be an intense physical experience. The climate, smells and tastes are different in the new country, and for some individuals everything comes together and induces a physical feeling of being out of place [30]. Ageing and illness can accentuate this feeling and, as Gardner points out, disease and age in itself are accompanied by ‘dis-ease’, the experience of not feeling comfortable: “*Sick bodies are bodies out of place”* [30] (p. 37). A way to overcome this uncomfortable feeling of being out of place is to move physically away from the place that induces the dis-ease.

A central aspect of the informants’ feelings of being in place upon their return to Bosnia was their ability to live close to their relatives. Since most return migrants stayed close to their family they could draw on their children’s care and help when necessary, and many were able to help care for their grandchildren, so that their children also benefitted from having their parents around. The significance of being part of inter-generational relations and the mutual need for help is also described by Vullnetari in her study of the lives and realities of elderly Albanians who had remained in Albania while their children had left for Greece as labour migrants. She found that not only did the older Albanians miss their children desperately and lacked their help with household chores and personal safety, their children also lacked carers for their own children. The traditional “care chain”, in which grandparents look after the grandchildren while the children are out at work, and the children take care of their ageing parents and help them with practicalities, had been broken as a consequence of the labour migration [31]. Along similar lines, Izuhara, in a study of Japanese migration, presents the concept of a “generational contract of family reciprocity” that consists of a continuous chain of obligations over generations. If parents and children do not live together, this contract is broken. Izuhara describes how Japanese women who have migrated to the UK move back to Japan when their parents become old in order to take care of them and to avoid breaking the “contract”. In some cases the parents move to the UK in order to be close to the children, providing another way in which children can fulfil their obligations towards their parents [32].

The fact that it would reflect badly on the children if the parents went into a nursing home emphasizes the importance of obeying the unspoken rule of the care chain. If the children neglect their responsibility towards their parents, both parents and children will be affected—the parents by a lack of care, and the children by social stigma. The importance of maintaining the care chain stresses the family’s role in caring. In agreement with this, one questionnaire-based study found that fewer than 40% of immigrants living in Denmark found it desirable to spend their old age in Denmark. This study also reported that the participants explained their unwillingness to live in a Danish nursing home mainly through the notion that welfare services should not replace the family in old age [33]. This point of view seems to be shared by the Bosnian informants. Taking care of family members who need help is a responsibility all family members have; it is not a responsibility the state should assume. 

A key issue in all the interviews, motivating return migration among elderly, chronically ill Bosnians, was the quest for a sense of belonging. It can be argued that a sense of belonging lies at the root of all well-being and vice versa, and it is therefore natural that it was an essential motive for return migration. In a quantitative study, Correa-Velez *et al*. found that the presence of a sense of belonging was the strongest predictor of mental well-being among young people with refugee backgrounds in Australia [34].

Feelings of belonging are not only connected to certain places; they are also strongly associated with certain people [35]. Accordingly, feeling socially embedded by being near their children and having social relations close by was described as being strongly associated with a sense of belonging among informants. This finding is shared by Kristiansen *et al.*, who studied attitudes towards return migration among immigrant women from Somalia, Turkey, India, Pakistan and Iran living in Denmark. Most of the informants in that study explained that they did not want to return to their country of origin because they had their families in Denmark [36]. One result of the impact of social ties, however, may be that the development of transnational communities and transnational families enables people to feel at home in several different locations [37]. This was also the case for the Bosnian refugees who had returned to Bosnia, many of whom had fond memories of their lives in Denmark. However, their feeling of belonging was indisputably stronger in Bosnia than in Denmark or anywhere else, due to the combination of close social relations and locations that had particular significance as places where they had been born and had grown up. This gave them a special feeling of home that provided them with mental well-being.

### Strengths and Limitations of the Study

This multi-sited study provides new knowledge in an area where many aspects have been left unexplored, and it is based on a relatively large number of interviews. By interviewing both returnees and non-returnees, we have obtained valuable insight into the background to opposite decisions with regard to return: we acquired more nuances and a deeper understanding of the drivers of return migration than we would have acquired by only interviewing one of the groups. 

Since the study was multi-sited and captured the temporal aspects of changes in well-being, we were able to show the influence of place and time on perceptions of health. Since the WHO definition of health has been criticized for being subjective, context-dependent and difficult to operationalize [38,39], its use as a framework for the present analysis can be contested. We are aware of the overlap and close interrelationship between the physical, social and mental aspects of well-being. We further acknowledge that the separation of drivers according to the type of well-being do not give due attention to the situatedness and intertwined nature of the different notions of health. However, we find the WHO definition useful in that it brings out nuances in perceptions of health that would not otherwise be revealed.

Using the snowball method to recruit participants might have resulted in the latter being more similar with regard to life stories, ideas and choices than would have been the case if a more randomized approach had been used. The choice of conducting individual interviews and interviews with couples instead of focus-group discussions was motivated by the adoption of a life-history approach, which necessitates in-depth interviews with individuals rather than structured interviews with groups of individuals [40]. We prioritized depth rather than breadth in our approach. 

The limitations connected to the use of an interpreter in interview situations are widely described in the literature [41]. We acknowledge that nuances generally may have been lost, and interviews where many family members were present were complicated to facilitate. However, we also acknowledge the positive effect on cultural sensitivity provided by the presence of a Bosnian interpreter acquainted with the local culture in this relatively brief study. Further, the fact that most interviews, whether conducted in Bosnian or Danish, were fully transcribed limited the loss of nuances.

## 5. Conclusions

The present study has explored why elderly, chronically ill Bosnian refugees return to Bosnia, as well as the role health issues play in the decision whether or not to return. Our findings show that social and cultural concerns constituted the predominant motives for return and that easy access to health-care services and medicine, and thus good physical health and the absence of illness, were not highly prioritized. It may therefore be tempting to conclude that health was not an important issue. However, if health is regarded more broadly as involving more than just physical health and the absence of illness, it can be argued that health *did* matter. Viewed as physical, social and mental well-being in line with the WHO definition, health was indeed one of the most important factors when the decision on return migration was made. 
